# When should RWE studies be prioritized for reimbursement? Insights from the Canadian perspective

**DOI:** 10.1017/S0266462325103401

**Published:** 2026-01-09

**Authors:** Jennifer Boss, Laura Shulak, Trisha Rao, Candice Tam, Shannon M. Sullivan

**Affiliations:** 1 PPD Evidera Health Economics & Market Access, Thermo Fisher Scientific, UK; 2 PPD Evidera Health Economics & Market Access, Thermo Fisher Scientific, Canada; 3 PPD Evidera Health Economics & Market Access, Thermo Fisher Scientific, France

**Keywords:** HTA, reimbursement, real-world evidence, pharmaceuticals, health policy

## Abstract

Given the increasing importance of real-world evidence (RWE) in health technology assessment (HTA) decision-making, we aimed to assess RWE use in Canadian HTA and collect stakeholder insights on when RWE generation should be prioritized in HTA.

We found that RWE was included in one-third of Canadian Drug Agency–L’Agence des médicaments du Canada (CDA-AMC) reimbursement reviews (2017–2022). To further understand drivers of RWE generation for reimbursement, consultations were held with stakeholders (pharmaceutical industry representatives, payers, and patient advocates) to obtain insights in Canada and other global markets. Stakeholders highlighted the value of RWE to complement randomized controlled trials (RCT) data, as well as the need to consider feasibility and multiple stakeholder perspectives.

Our findings indicate there is a need to further support the practical implementation of RWE in policy decision-making. A framework providing guidance on when to prioritize RWE studies for reimbursement would provide value and could be tailored by region.

## Overview of RWE in reimbursement

While randomized controlled trials (RCTs) are the gold standard for assessing efficacy and safety of treatments in regulatory decision-making, they are limited in their ability to provide evidence of an intervention’s value in a real-world context (1). Real-world evidence (RWE), generally defined as observational evidence derived outside RCTs, can fill data gaps and inform reimbursement decisions (2;3). Canada aims to be one of the first jurisdictions to implement RWE to support RCTs in informing health technology assessment (HTA)/reimbursement (4). In 2023, the Canadian Drug Agency–L’Agence des médicaments du Canada (CDA-AMC) partnered with Health Canada and Institut national d’excellence en santé et services sociaux to create the Guidance for Reporting Real-World Evidence, which promotes RWE standardization by providing core conduct and reporting guidance for RWE studies being submitted for reimbursement (5).

However, uncertainty remains around *when* RWE generation should be prioritized and conducted for reimbursement. While many health technologies are coming to market, not all will benefit equally from RWE to optimize patient access. RWE studies are time-consuming and require considerable resources. Therefore, judicious RWE generation planning is essential during asset development to ensure that once approved, access is optimized to meet patient and health-system needs.

This article outlines our efforts to determine how and when RWE is used for reimbursement. We did this by identifying past CDA-AMC reimbursement reviews that included RWE to understand *how RWE has been considered in past Canadian HTA.* We also collected insights from key Canadian, regional, and global stakeholders, including pharmaceutical industry representatives, payers, and patient advocates, on *when RWE generation should be prioritized for reimbursement.* We integrated our findings to identify common themes around when it is impactful to generate RWE for reimbursement and barriers/facilitators to generating these data. Details on methodology are presented in the Supplementary Methods.

## Use of RWE in Canadian reimbursement reviews

We reviewed 379 CDA-AMC reimbursement submissions from 2017 to 2022 and found that RWE was included in approximately one-third of submissions (*n* = 145). No clear trends were identified in the inclusion of RWE over the 6 years. The most common therapeutic areas for RWE inclusion were oncology (50 percent), genetic disorders (10 percent), and neurology (9 percent). Forty-three percent of reviews for rare diseases included RWE.

We conducted a detailed data extraction on a random sample of 76 reviews with RWE to assess the RWE’s characteristics, purpose, and impact (i.e., whether it was discussed in any level of detail in the report) and influence (i.e., whether it was mentioned in the final recommendation report, regardless of the decision). RWE was most often generated to fill gaps in economic model inputs (27 percent), comparative efficacy evidence (18 percent), efficacy and safety data (14 percent), and to assess the validity of outcome measures (8 percent).

We found large variability in the impact of RWE on decision-making based on a preliminary analysis of 11 reimbursement reviews from 2022. There was a lack of correlation between whether the CDA-AMC committee discussed the RWE beyond simply including the evidence in the report (e.g., highlighting limitations in methodology and raising concerns about generalizability to the Canadian population) and if the RWE was deemed influential on the final recommendation. While the lack of correlation prevents making robust conclusions on the role of RWE in influencing decisions, the inclusion of even low-quality RWE (e.g., small sample size and risk of bias or confounding due to the study design) suggests that these data may still influence decision-making if they represent the best available evidence.

Several analyses of HTA reimbursement reviews containing RWE have been published (6–10). However, compared to our analysis, most of these studies had a more focused scope (e.g., oncology drugs only, use of a specific database, and use of an external control arm) or aimed to answer a different research question.

## Stakeholder insights on the prioritization of RWE for reimbursement

**Figure 1.**
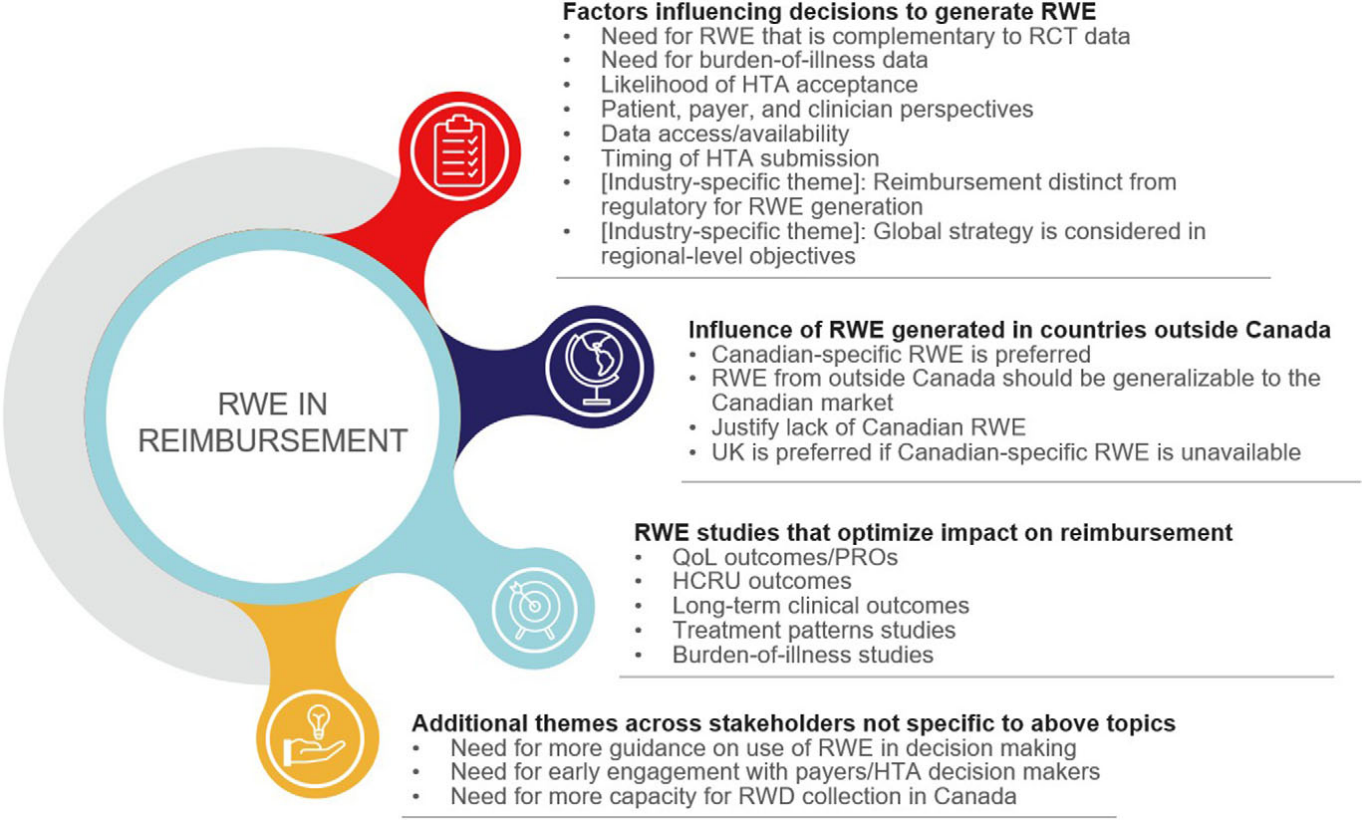
Key themes from stakeholder insights by topic. Abbreviations: HCRU, healthcare resource utilization; HTA, health technology assessment; PRO, patient-reported outcome; QoL, quality of life; RCT, randomized controlled trial; RWD, real-world data; RWE, real-world evidence.

We next sought to draw upon the expertise of different stakeholder groups to identify key learnings and current practices around generating and prioritizing RWE studies for reimbursement in Canada and globally. We conducted 1-1 interviews with a total of 13 stakeholders; 3 prespecified topics were discussed: (a) what factors influence decisions to conduct RWE studies, (b) when RWE generated in other countries influences this decision, and (c) when RWE can optimize reimbursement. Based on a thematic analysis of the interviews, the stakeholders agreed on several themes related to each prespecified topic, as well as additional themes that emerged during the interviews (Figure 1; Table 1).

**Table 1.** Key themes from stakeholder insights by topic, with theme descriptions and representative stakeholder quotes

Theme	Theme description
**Factors influencing decisions to generate RWE**	
Need for RWE that is complementary to RCT data	RWE alone is not sufficient for reimbursement; however, RWE can add value by being complementary to RCT data (e.g., validation of RCT results and exploration of RCT outcomes in a larger/different population)
Need for burden-of-illness data	RWE studies can generate burden-of-illness data that are important for reimbursement (i.e., epidemiology, economic model inputs, and insight into unmet need)
Likelihood of HTA acceptance	Stakeholders consider how likely HTA bodies are to accept RWE
Patient, payer, and clinician perspectives	Stakeholders consider different stakeholder perspectives in RWE generation, including patients, clinicians, and payers
Data access/availability	Data access and availability impact the feasibility to generate RWE, and to generate RWE in time for HTA submission
Timing of HTA submission	
[Industry-specific theme]: Reimbursement distinct from regulatory for RWE generation	RWE generated for reimbursement may be distinct from that generated for regulatory approval, but may require alignment across cross-functional teams
[Industry-specific theme]: Global strategy is considered in regional-level objectives	Regional affiliates consider the global team’s strategy for RWE generation when developing local RWE evidence generation plans
**Industry stakeholder:** *“Data access I think is definitely a challenge in Canada, especially when you’re looking at the provinces, there are certain provinces where it is a lot easier to get access to data, whereas in other provinces it’s a lot more difficult. Data access is another factor that we have to consider.”*	
**Payer stakeholder:** “*You can’t use RWE to supersede RCT trial data in any population…You can use it to complement your value proposition but never supplant.*”	
**Patient advocate stakeholder:** “*Up to a number of years ago the patient voice was never heard at these meetings. When you went to the approval, HTAs, the patient wasn’t there, the patient advocate wasn’t there.”*	
**Influence of RWE generated in countries outside Canada**	
Canadian-specific RWE is preferred	Canadian-specific RWE is preferred for reimbursement
RWE from outside Canada should be generalizable to the Canadian market	If not available or feasible to generate Canadian-specific RWE, then RWE used from outside of Canada should be generalizable to the Canadian market
Justify the lack of Canadian RWE	Justification should be provided to HTA bodies for why Canadian RWE is not being utilized
The UK is preferred if Canadian-specific RWE is unavailable	RWE from the UK is generally preferred for RWE from outside of Canada based on the similarities in healthcare systems and patient access
**Industry stakeholder:** *“We look at things like burden-of-illness, so we try to understand what the disease looks like in Canada, the treatment landscape…and then any sort of evidence generation…If we’re trying to get a product reimbursed, we want to try to understand the treatment landscape.*”	
**Payer stakeholder:** *“I do care about consistency and decision-making with other cost-effectiveness countries.”*	
**Patient stakeholder:** *“My experience with this disease, can be totally different to somebody else with the exact same disease, and if you don’t get a broad enough pan-Canadian view of what the challenges are and what the disease impacts are, you’re missing a big piece of the story.”*	
**RWE studies that optimize impact on reimbursement**	
QoL outcomes/PROs	These RWE study types and outcomes are those that could have the greatest impact on reimbursement decision-making
HCRU outcomes	
Long-term clinical outcomes	
Treatment patterns studies	
Burden-of-illness studies	
**Industry stakeholder:** *“When you have tons of clinical uncertainty or your trial hasn’t [been] conducted [with] that type of evidence, RWE is incredibly powerful.”*	
**Payer stakeholder:** *“QoL in Canada is not a decision criterion, it is an enabler. Run an RWE, show it maintains [QoL], that’s better than having nothing.”*	
**Patient stakeholder:** *“We can look at hospitalizations and visits and other kinds of medications… But from a patient point of view, it’s also that experience of… how am I feeling today? Did I get out of bed?…what’s important to patients is [how] do I function on a daily basis? How do I feel on a daily basis as opposed to some of the other measures that…may or may not be really directly impactful on me or they happen…so infrequently.”*	
**Additional themes across stakeholders not specific to the above topics**	
Need for more guidance on the use of RWE in decision-making	More guidance on RWE for reimbursement is needed; the CDA-AMC guidance on RWE focuses on methodology, rather than on how the data will be used and interpreted in decision-making
Need for early engagement with payers/HTA decision-makers	Early engagement with payers and HTA bodies is important for identifying relevant evidence gaps that can be filled by RWE and for gaining insight into optimal study design
Need for more capacity for RWD collection in Canada	Canada could benefit from networks that centralize RWD collection across provincial/territorial jurisdictions (i.e., pan-Canadian) to increase efficiencies in RWE generation, including facilitating patient participation in RWE and improving data sharing
**Patient stakeholder quote on additional theme on “Need for more guidance on RWE”:** *“A lot of the guidelines that came out were really around the validity, robustness of the evidence being generated, but not really what evidence and…where and how that evidence would be used.”*	
**Payer stakeholder quote on additional theme on “Need for early engagement with payers/HTA decision makers”:** *“I think the disconnect is, you still haven’t connected the payer with the vendor in a constructive dialogue to actually strategize the use of RWE early on in the cycle.”*	
**Industry stakeholder quote on additional theme of on “Need for more capacity for RWD collection in Canada:** *“I hope one day we’ll see full pan-Canadian data that can be used and shared…there must be a way because the country is rich in RWE data.”*	

Abbreviations: CDA-AMC, Canadian Drug Agency–L’Agence des médicaments du Canada; HCRU, healthcare resource utilization; HTA, health technology assessment; PRO, patient-reported outcome; QoL, quality of life; RCT, randomized controlled trial; RWD, real-world data; RWE, real-world evidence; UK = United Kingdom.

All stakeholders were aligned on the value RWE can provide as complementary evidence to support RCT data, to fill evidence gaps left by RCTs, or to validate RCT results in larger and/or different populations; the theme “need for RWE that is complementary to RCT data” had high concordance (defined as ≥8 stakeholders) in our analysis.

There was general agreement among all stakeholders that certain types of RWE studies are usually more important for reimbursement purposes, specifically those characterizing patient-reported outcomes, current treatment patterns, healthcare resource utilization, and long-term clinical outcomes (Figure 1). RWE can be used to reduce uncertainty around these topics by establishing existing disease burden and addressing evidence gaps from RCTs.

For reimbursement in Canada, stakeholders agreed that Canadian data is preferred, but they believe data availability limits the feasibility of generating such evidence (the theme “data access/availability” had high concordance). This belief echoes an analysis of stakeholder perspectives in Canada, which identified inadequacies in real-world data (RWD) infrastructure and access as the greatest barrier to using RWE for decision-making for cancer drugs (11). Canadian stakeholders in our analysis stated that when not possible, justification should be provided for the use of data from outside of Canada, including generalizability to the Canadian population (5). From our assessment of CDA-AMC reimbursement submissions, manufacturers rely heavily on RWD from other countries, with more than half of reviews using RWE from other regions (*n* = 24 out of 43 reviews that reported the region[s] for all included RWE studies). Other regions frequently represented included the United States, United Kingdom, and France, which aligned with Canadian stakeholders’ insights, indicating the importance of similarities in healthcare systems. Canadian patient advocate stakeholders noted that for rare diseases, consolidating data from other markets, and particularly the United States, may be a reasonable approach to obtaining RWD rather than focusing only on Canadian data. Stakeholders with global experience also cited a preference for local data, followed by data that are generalizable to the region of interest.

Industry stakeholders considered the likely acceptability by HTA bodies and payers when making decisions about RWE generation and noted this may vary by region. Trends in RWE acceptance in Europe were explored in a recent survey, which found that European Network for Health Technology Assessment member HTA organizations were more likely to accept RWE for orphan drugs and when there is a companion diagnostic, and were least likely to accept non-local RWE, although the impact on decision-making was not assessed (12). As we found in our analysis, some stakeholders highlighted that early engagement with HTA bodies and payers can identify data gaps that RWE can fill and opportunities to optimize RWE study design. Payer stakeholders emphasized that RWE should be robust and generated in studies conducted with high methodological rigor.

Incorporation of the patient perspective in RWE was deemed important by Canadian patient advocate stakeholders as they believe these data provide valuable insight into the patient and caregiver experience – insight that until recently had not been routinely considered in reimbursement decision-making. However, they emphasized that low patient involvement is a challenge and suggested providing patient education around the benefit of RWE study participation and involving patients in early RWE generation planning to encourage engagement. Involving patients in initiatives to support RWE incorporation in cancer drug HTA in Canada was previously recommended by stakeholders in a 2020 study (11).

Our thematic analysis highlighted that the majority of stakeholders recognized that the value of RWE lies in its ability to complement RCT data, and that RWE is typically not sufficient to support reimbursement on its own. Furthermore, most stakeholders discussed the need to consider feasibility, as well as the perspectives of different stakeholders to optimize RWE for reimbursement.

## Limitations

A key limitation of the analysis of CDA-AMC reimbursement reviews was variability in how RWE was described in the CDA-AMC reports, which may have led to undercounting the reviews with RWE if not clearly documented. Detailed analysis was conducted only on a small random sample of CDA-AMC reviews, and the resulting data may not be representative of larger datasets. In addition, our study only used RWE information included in the CDA-AMC reports and did not assess the cited RWE studies in depth.

The stakeholder insights research was a small sample. Additionally, due to fewer patient advocates and payer stakeholders, results may be biased toward the industry perspective. Our study was designed to elicit preliminary insights from a sample of stakeholders. As such, the insights presented are high-level and may not be representative of all stakeholders of a given type or region.

## Gaps and potential solutions

Key gaps identified in our analysis include a lack of guidance on the practical implications of RWE in reimbursement decision-making, as well as challenges in RWD availability, access, and collection in Canada. The recent guidance published by CDA-AMC encouraging robust design of RWE studies, and the general preference of HTA agencies for high-quality data, may contribute to greater acceptability of RWE in Canada; however, these guidelines – as well as recent global RWE guidelines – are heavily focused on methodology (5). Developing a methodologically robust, practical framework that could be tailored to different regions, helps stakeholders prioritize when to generate RWE for reimbursement, and incorporates stakeholder perspectives would provide value. Given that HTA bodies are increasingly including non-payer stakeholders in decision-making, earlier involvement of such stakeholders in RWE planning could better inform manufacturers and facilitate the inclusion of valuable perspectives. Additionally, greater engagement in scientific advice programs offered by HTA agencies could increase the impact of RWE (13–16).

Although Canadian-specific data would be preferred for generalizability of RWE, access to and collection of Canadian data vary across provincial/territorial jurisdictions, making it difficult for manufacturers to use for RWE. This suggests a need for cross-country collaboration among data managers to build and maintain pan-Canadian RWD sources (17). Several recent initiatives have launched within Canada (such as the Pan-Canadian Real-World Health Data Network) (18–20), all of which focus on consolidating available real-world datasets in a central database for easy accessibility. Such offerings could increase RWE generation and have valuable downstream uses in reimbursement decision-making. Improvements in RWE generation capabilities would improve data quality and, thus, enable RWE to be more influential in HTA decisions.

## Key takeaways

Taken together, our analysis of CDA-AMC reimbursement reviews indicates that while RWE was included in some submissions, presumably representing the best available evidence in those cases, it was not commonly used in Canadian HTA during the period of analysis. Only approximately one-third of submissions included in our analysis included RWE, indicating there may be an opportunity for greater utilization of RWE in reimbursement decision-making in Canada.

Our research highlights important areas to consider when prioritizing RWE studies for reimbursement. RWE should primarily be used as complementary evidence to support, validate, or fill gaps left by RCTs. Current guidance provided globally by HTA bodies and regulatory agencies focuses on RWE methodology, but there is a need for further practical guidance, such as a robust decision framework to support optimized RWE generation for reimbursement.

## Supporting information

10.1017/S0266462325103401.sm001 Boss et al. supplementary material Boss et al. supplementary material

## Supplementary material

The supplementary material for this article can be found at http://doi.org/10.1017/S0266462325103401.
