# Supplementary material for: When should RWE studies be prioritized for reimbursement? Insights from the Canadian perspective
Source: Int J Technol Assess Health Care. 2026 Jan 9;42(1):e19. doi: 10.1017/S0266462325103401 (PMC12964149; doi:10.1017/S0266462325103401)
Supplement: Boss et al. supplementary material [file S0266462325103401sup001.docx]

**Full Title**: When should RWE studies be prioritized for reimbursement? Insights from the Canadian Perspective

**Running Title**: RWE In Reimbursement Decision-Making

**Authors**:

Boss, Jennifer; MSc; PPD Evidera Health Economics & Market Access, Thermo Fisher Scientific, London, UK

Shulak, Laura; MSc; PPD Evidera Health Economics & Market Access, Thermo Fisher Scientific, St-Laurent, QC, Canada

Rao, Trisha; PhD; PPD Evidera Health Economics & Market Access, Thermo Fisher Scientific, St-Laurent, QC, Canada

Tam, Candice; MSc, MPharm; PPD Evidera Health Economics & Market Access, Thermo Fisher Scientific, London, UK

Sullivan, Shannon M; MSc, PhD; PPD Evidera Health Economics & Market Access, Thermo Fisher Scientific, Ivry-sur-Seine Cedex, Île-de-France, France

Corresponding Author: Jennifer Boss
Address: The Ark, 2^nd^ Floor, 201 Talgarth Road, London, W6 8BJ, United Kingdom
Mobile: +44 75 4543 8185
Email: jennifer.boss1@thermofisher.com

# Supplementary Methods

## Analysis of RWE in CDA-AMC Reimbursement Reviews

Analyses identifying RWE use in Canadian Drug Agency–L’Agence des médicaments du Canada (CDA-AMC) reimbursement reviews between 2017 and 2022 were conducted. Basic information on the review characteristics (e.g. drug name, therapeutic area, reimbursement recommendation, type of submission) was extracted into a Microsoft® Excel spreadsheet for all reimbursement review reports identified during the six-year period. Each report was searched for pre-specified terms related to RWE (i.e., ‘real-world’, RWE, real-world data [RWD], observational, non-randomized, uncontrolled, observational, retrospective, prospective, cohort, registry/registries), and those that included one or more terms were included regardless of the RWE source, study characteristics, or outcomes. Submissions that only cited RWE but did not describe the study(ies) in the report (e.g., search terms appeared only in the bibliography) were not included. Specific data extraction variables of basic information related to RWE was conducted for all included reimbursement reviews. Following this, fifty percent of the reviews with RWE were randomly selected for further data extraction (including variables on the RWE characteristics such as study objective, design, region, sample size, and conclusion).

Analysis of the impact of RWE included in the review was assessed in terms of the following: (1) the gap the RWE was intending to fill; (2) how the RWE was critiqued (defined as limitations or criticisms of the RWE from the CDA-AMC committee); and (3) whether the RWE influenced the final recommendation (for our analysis, we considered RWE influential if it was mentioned in the final recommendation report regardless of the decision). These assessments were conducted on all reviews that included RWE from 2017 to 2022, except whether the RWE influenced the final recommendation, which was conducted on a subset of 11 reviews from 2022 to examine the most recent reviews from the overall dataset, and gain insights into trends in RWE use in reimbursement decisions that would be as current as possible.

Descriptive statistics were generated for the total numbers of reimbursement reviews and proportions based on selected characteristics of the reviews (e.g., year recommendation issued, therapeutic area, region) or the impact of the RWE studies included in the reviews (e.g., gap the RWE was intending to fill).

## Data Collection from Stakeholder Insights

Consultations with stakeholders (pharmaceutical industry representatives, payers, and patient advocates) were conducted to obtain insights in Canada and other global markets on three pre-specified topics: (a) what factors influence decisions to conduct RWE studies, (b) when RWE generated in other countries influences this decision, (c) when RWE can optimize reimbursement. Stakeholders who were interested in participating in the study were first provided with a slide deck overview of early work conducted on the analysis of RWE in CDA-AMC reimbursement reviews, after which an educational meeting was held where the authors presented an overview of the research objectives and stakeholder consultations. Following the educational meeting, if stakeholders agreed to participate further, a second 1-1 meeting was held to consult on RWE and its impact on reimbursement.

Stakeholder consultations were conducted between June 2023 and January 2024 and were held virtually via Microsoft® Teams, lasting between 30 and 60 minutes. Two researchers from our author team attended each meeting. Transcribing of the consultations was conducted by an author who had not attended the meeting, and then validated by one or both of the authors who had attended the meeting. The transcribed notes were anonymized and analyzed based on an adaptation of the methods used by Farmer et al. 2023 (i.e., framework analysis) (1). Stakeholder responses were reviewed and synthesized into themes that were categorized into each of the three pre-specified topics on RWE and its impact on reimbursement. Key insights from the consultations were mapped into a Microsoft® Excel spreadsheet that allowed for categorization by topic and comparison across stakeholders; an insight common across two or more individual stakeholders was designated as a theme, which was validated by a second author. Finally, anonymized quotes were selected to highlight each theme and to illustrate nuances of a particular stakeholder’s insight.

# References

1. Farmer C, O'Toole B, Barnish MS, Trigg LA, Hayward S, Crathorne L, et al. Early access schemes for innovative health technologies: the views of international stakeholders. *Int J Technol Assess Health Care*. 2023 Jul 6;39(1):e45.
